# Artificial Intelligence (AI) Assessment of Pediatric Dental Panoramic Radiographs (DPRs): A Clinical Study

**DOI:** 10.3390/pediatric16030067

**Published:** 2024-09-11

**Authors:** Natalia Turosz, Kamila Chęcińska, Maciej Chęciński, Karolina Lubecka, Filip Bliźniak, Maciej Sikora

**Affiliations:** 1 Department of Maxillofacial Surgery, Hospital of the Ministry of Interior, Wojska Polskiego 51, 25-375 Kielce, Poland; 2 Department of Glass Technology and Amorphous Coatings, Faculty of Materials Science and Ceramics, AGH University of Science and Technology, Mickiewicza 30, 30-059 Kraków, Poland; checinska@agh.edu.pl; 3 Department of Oral Surgery, Preventive Medicine Center, Komorowskiego 12, 30-106 Kraków, Poland; maciej@checinscy.pl (M.C.); lubeckarolina@gmail.com (K.L.); fblizniak@gmail.com (F.B.); 4 Department of Biochemistry and Medical Chemistry, Pomeranian Medical University, Powstańców Wielkopolskich 72, 70-111 Szczecin, Poland

**Keywords:** dental caries, artificial intelligence, panoramic radiography, DMF index

## Abstract

This clinical study aimed to evaluate the sensitivity, specificity, accuracy, and precision of artificial intelligence (AI) in assessing permanent teeth in pediatric patients. Over one thousand consecutive DPRs taken in Kielce, Poland, with the Carestream CS9600 device were screened. In the study material, 35 dental panoramic radiographs (DPRs) of patients of developmental age were identified and included. They were automatically evaluated with an AI algorithm. The DPRs were then analyzed by researchers. The status of the following dichotomous variables was assessed: (1) decay, (2) missing tooth, (3) filled tooth, (4) root canal filling, and (5) endodontic lesion. The results showed high specificity and accuracy (all above 85%) in detecting caries, dental fillings, and missing teeth but low precision. This study provided a detailed assessment of AI performance in a previously neglected age group. In conclusion, the overall accuracy of AI algorithms for evaluating permanent dentition in dental panoramic radiographs is lower for pediatric patients than adults or the entire population. Hence, identifying primary teeth should be implemented in AI-driven software, at least so as to ignore them when assessing mixed dentition (ClinicalTrials.gov registration number: NCT06258798).

## 1. Introduction

### 1.1. Background

Dental caries is one of the most prevalent diseases among children, caused by factors such as the frequent consumption of sugars, poor oral hygiene, and insufficient fluoride exposure [1,2]. Researchers are also looking for genetic factors predisposing to dental caries [3,4,5]. It develops more rapidly in deciduous teeth than in permanent ones, as their enamel is significantly thinner [6].

The decayed, missing, and filled teeth (DMF) index is an indicator used for the initial assessment of dental conditions among the population undergoing preventive dental visits or during the initial visit where dental treatment is planned. As the index can evaluate primary, mixed, and permanent teeth, it is useful for both children and adult patients [7]. For this reason, it is an important indicator for this study, as it focuses on this type of assessment of the changes in the teeth of pediatric patients. Originally, the DMF index values were determined using basic instruments during physical examination. However, if dental radiography is available, it may supplement physical examination. In this study, the classic DMF index was expanded to DMFRE to include the detection of root canal fillings (R) and endodontic lesions (E). This step was taken to make this study even more comprehensive and additionally check the efficiency of the AI algorithm in assessing these aspects. This may determine the direction of the development of algorithms in the assessment of dental radiographs in the near future.

The diagnosis of caries relies mainly on visual–tactile examination and imaging tests, including dental panoramic radiographs (DPRs) [8]. Various indicators assessing the epidemiology of dental caries are calculated based on the above examinations. DPRs are a valuable diagnostic tool, providing information about all teeth and the maxillofacial skeleton [9]. The quality of modern panoramic radiographs allows for assessing individual teeth and the condition of the periodontium, both marginal and periapical. However, identifying early caries lesions may be difficult, as only a 30–40% loss of enamel mineral will be detectable during radiological examination [10,11]. 

DPR examination involves radiation and generally requires indications. While performing a control panoramic radiograph every few years in adults seems to be justified, in the case of pediatric patients, it may be controversial. However, indications for DPRs in children do exist and include, among others, diagnoses in the field of orthodontics and dental surgery such as (1) development evaluation and teeth location, (2) bone lesions, (3) identification of foreign bodies, or (4) craniofacial trauma [12]. As DPRs provide a lot of information, not every dentist has enough time to thoroughly analyze radiographs and detect pathologies outside their specialty. It is reasonable to believe that every DPR should be evaluated and described by a dentist with appropriate training or a radiologist specializing in the evaluation of dental radiographs. However, the time and cost of such work significantly limit such an approach.

### 1.2. Rationale

Artificial intelligence (AI) is the ability of computers and machines to perform tasks that would typically require human intelligence or intervention [13]. Its application also includes the pediatric field, helping in diagnosing, e.g., pulmonary diseases, and improving neonatal daily care or fetal urology [13,14,15]. This technology has also been introduced in pedodontics in the automated charting of dental status, detecting permanent tooth germs, and classifying supernumerary teeth, including mesiodens [16]. Predicting early childhood caries with an AI-driven model can be helpful in identifying high-risk groups and implementing appropriate prevention [17]. The rapid advancement of artificial intelligence (AI) gives hope for automating the screening of DPRs [18,19]. This technology can be beneficial in providing results, even if it means overestimating issues (type I error), as it would motivate dentists to verify the diagnoses and reduce the risk of omissions. However, it is difficult to determine the general sensitivity of AI algorithms in assessing DPRs. Various aspects are analyzed separately, such as teeth identification and numbering, detecting caries, periapical lesions, or periodontal bone loss. There are already systematic reviews on this topic, and recently, an overview of such reviews has also been published [18]. It shows that AI rates are increasing year by year, which indicates the promising potential of this technology in the daily assessment of DPRs. Nevertheless, these findings refer to the radiographs of adult patients. AI faces some challenges in pediatric radiography, like paucity of data, partly due to ethical concerns and stricter regulations regarding data [20,21]. Radiographs may not be of high quality because of motion artifacts. Moreover, the presence of mixed dentition and the ongoing development of permanent teeth can significantly complicate the assessment of the algorithm. Overlapping images of primary and permanent tooth tissues, as well as the resorption of primary teeth caused by erupting permanent teeth, may pose additional difficulties for the still incipient algorithm. In addition, the shape of a tooth changes during development, so the algorithm needs to be trained for each stage of the development of each type of tooth (incisor, canine, premolar, molar). However, it is crucial to assess the effectiveness of AI in evaluating mixed dentition and developmental stages. No studies were found that used this technology for comprehensive DPR analysis in pediatric patients.

### 1.3. Aim

The aim of this clinical study was to determine the sensitivity, specificity, accuracy, and precision of an AI algorithm in the assessment of permanent teeth with incomplete development and mixed dentition in pediatric patients.

## 2. Materials and Methods

### 2.1. Study Design

This study was designed as a prospective double-gate trial of diagnostic accuracy. It aimed to calculate the sensitivity, specificity, precision, and accuracy of the AI algorithm by evaluating the same DPR twice—first by the AI algorithm and then by researchers. The performance metrics revealed a discrepancy between the human evaluation (reference test) and the test results of the algorithm analysis (index text). The study was conducted according to the guidelines of the Declaration of Helsinki and approved by the Świętokrzyska Medical Chamber in Kielce Bioethics Committee (2.3/2023 of 31 August 2023) and ClinicalTrials.gov (NCT06258798) [22,23]. The research report was prepared under the STARD 2015 protocol [24].

### 2.2. Research Material

Consecutive DPRs of developmental-age patients were included in the study. The detailed study inclusion and exclusion criteria were defined following the acronym PICOS (population, intervention, comparator, outcomes, and settings). They are presented in Table 1. The inclusion condition based on the population criterion was the presence of at least one permanent tooth with unfinished development, other than a wisdom tooth, in the DPR. Unfinished development was understood as any stage of crown and root development (from C_i_ to A_1/2_), before root apices closure (A_c_) [25].

According to the current overview, AI algorithms achieve the highest accuracy for the missing teeth variable, on average 93.67% [18]. The missing teeth identification sensitivity is 97% [18]. There are no specific data for pediatric patients. We assumed a 5% type I error, a prevalence of 9% estimated based on preliminary data, and a marginal error of 10% [26]. The required sample size calculated with a Sample Size Estimation for Diagnostic Accuracy Studies tool was 124 tests [27]. Due to probable differences between the values of variables for the individual 8 positions in the oral quadrant, we increased the sample size to 1120 tests for each variable (32 oral locations on 35 DPRs).

### 2.3. Test Methods

In the study, the provided AI-driven software was used to analyze DPRs taken with the Carestream CS 9600 device. An automated dental chart for each radiograph was obtained. Then, human analysis was performed. The reference point was the assessment of a 4-year experienced doctor of dental surgery and engineer (N.T.). In case of doubt, a doctor of dental surgery and maxillofacial surgeon with 12 years of experience had an advisory vote (M.C.). To ensure inter-rater reliability, rater training, standardization of procedures, pilot tests, and calibration sessions were undertaken. Both investigators completed the same dedicated radiograph assessment training organized by the Polish Supreme Medical Chamber. Then, they conducted pilot radiograph assessments using the AI algorithm discussed in this study. Based on a detailed analysis of the algorithmic assessment results, they standardized the human assessment procedures. Then, pilot tests were performed, and several calibration sessions (including the use of external reference images) were performed to eliminate differences in the interpretation of the same radiological image.

The sensitivity, specificity, accuracy, and precision (Table 2) of the AI algorithm were calculated, which are typically reported to summarize the performance of a model [28]. They are often used to evaluate algorithms performing DPR analysis [18]. The choice of variables resulted directly from the capabilities of the tested algorithm. The status of the following dichotomous variables was assessed: (1) decay—the presence of at least one defect in the crown of a permanent tooth, (2) missing—the absence of a permanent tooth in a given position in the oral cavity, (3) filled—the presence of at least one filling in a permanent tooth, (4) root canal filling—the presence of at least partial filling in the chamber or root canal of a permanent tooth, (5) endodontic lesion—the presence of radiolucency covering the apex of at least one of the roots of a permanent tooth. A conclusive result was understood as a confirmation or denial of a given diagnosis (decay, missing tooth, filling, etc.). The algorithm used did not provide other, i.e., inconclusive, results. The lack of a decision by the researchers regarding the diagnosis in a given location of the oral cavity was defined as an inconclusive result.

### 2.4. Analyses

The agreement between the AI and human assessment results was analyzed for all patients, all 32 oral positions corresponding to the natural positions of permanent teeth, and all five Boolean variables discussed above. The sensitivity, specificity, accuracy, and precision of the measurements depending on the variable and position in the oral cavity were calculated. In addition, the Pearson’s correlation coefficients of false-positive and false-negative results with the presence of a primary tooth in a given position in the oral cavity were calculated. The absolute values of the coefficient were interpreted as follows: (1) 0.00–0.09—negligible correlation; (2) 0.10–0.39—weak correlation; (3) 0.40–0.69—moderate correlation; (4) 0.70–0.89—strong correlation; (5) 0.90–1.00—very strong correlation.

The results were presented in tables and charts. Google Sheets (version 2024.05.31, Google LLC, Mountain View, CA, USA) and MedCalc software (version 23.0.1; MedCalc Software Ltd., Ostend, Belgium) were used [29].

## 3. Results

### 3.1. Sample Tested

In the study, 1021 consecutive DPRs were screened. All these radiographs were taken using the device Carestream CS9600 at a radiology department located in Kielce, a city in southern Poland, between September 2022 and June 2023. Most of the radiographs (97%) were rejected at the screening stage as non-pediatric. As a result, the DPRs of 35 patients of developmental age (16 females and 19 males) were included in the study. None of the initially qualified radiographs were excluded during further evaluation. There were no algorithm nor human errors resulting in missing or partial results. The average age of participants was approximately 10 years old (SD = 2.2, Range = 10).

### 3.2. Test Results

**Figure 1:**
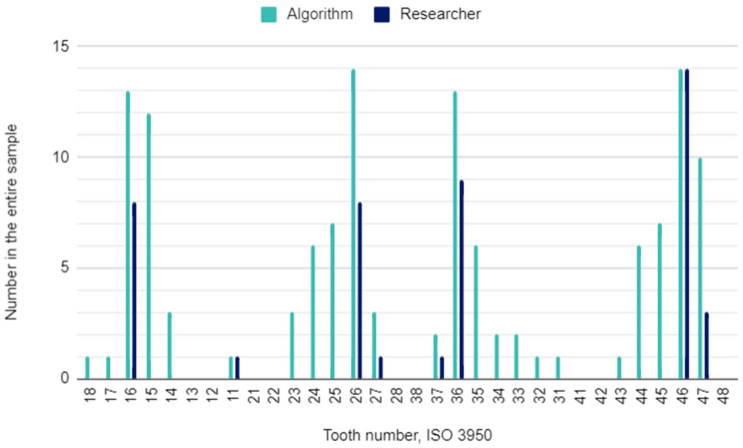
Decayed teeth by their location in the oral cavity.

**Figure 2:**
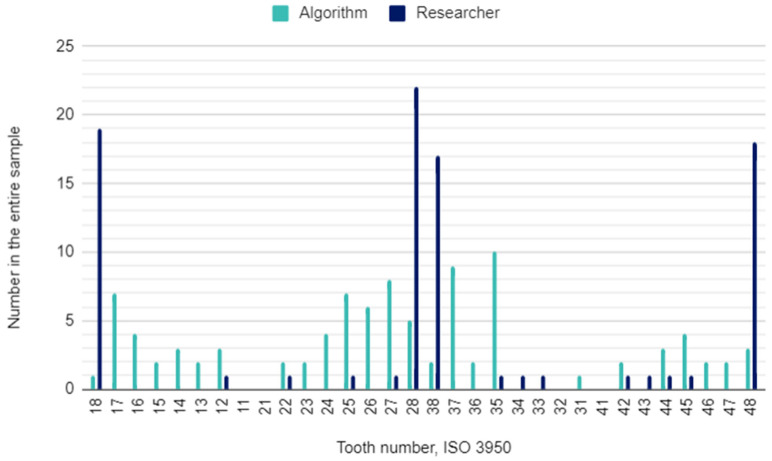
Missing teeth by their location in the oral cavity.

**Figure 3:**
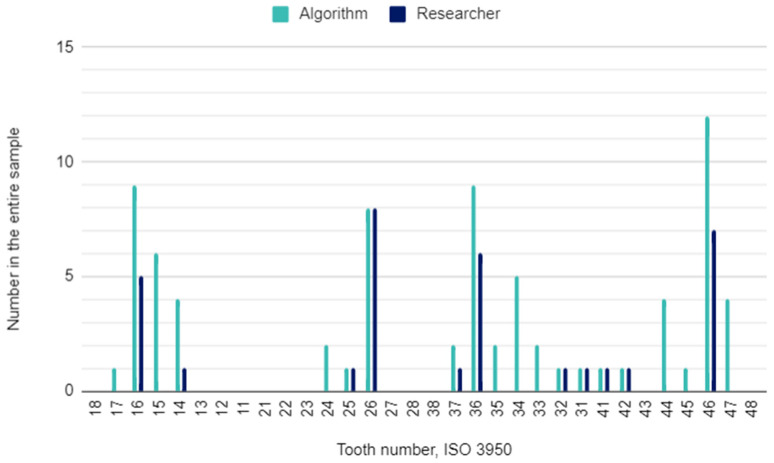
Filled teeth by their location in the oral cavity.

The principal investigator assessing DPRs had concerns in 22 (2%) of 1120 oral locations. In these cases, the initial assessment was verified by the other researcher. The raw results for individual variables and the results of the calculations of the sensitivity, specificity, accuracy, and precision of the AI algorithm are presented in Table 3. This table is a collective presentation of all tests with content corresponding to the STARD 2015 flow diagram [24]. The number of potentially eligible participants was equal to the eligible participants’ number and the tested participants’ number and was 35. There was no case of a missing reference standard. There were no inconclusive test results for either AI or human evaluation. The numbers of teeth identified by the AI and investigators for each diagnosis are presented in Figure 1, Figure 2 and Figure 3.

Based on the researchers’ assessment, the examined material revealed 224 primary teeth. This means that, statistically, deciduous teeth were present in 20% of the examined positions in the oral cavity. Therefore, there were, on average, 6.4 primary teeth per DPR. The correlations between a false-positive or a false-negative response in the index test and the presence of a primary tooth in a given position in the oral cavity were calculated. The results are presented in Table 4. None of them were strong. When assessing decayed and missing teeth, a slight positive correlation with false-positive results and a slight negative correlation with false-negative results is noteworthy. For the remaining variables, no correlation of the AI error with the presence of a primary tooth in a given position in the oral cavity was observed.

## 4. Discussion

The accuracy of artificial intelligence algorithms in evaluating permanent teeth in dental panoramic radiographs is lower for pediatric patients than for adults or the general population, showing high specificity but low precision.

### 4.1. Decayed Teeth

The results of the caries assessment by AI in this study are worth comparing with the results of two systematic reviews by Mohammad-Rahimi et al. and Prados-Privado et al., who reviewed deep learning studies on radiological caries detection [30,31]. In the first review, the average specificity of the analyzed models was 86%, precision was 84.97%, accuracy was 89.67%, and sensitivity was not assessed [30]. In the second review, only accuracy was assessed, and it reached 86% [31]. The results of our study are, therefore, comparable to the specificity and accuracy of the abovementioned reviews, except for a significant difference in precision (26.36% in the current study versus 84.97% in Mohammad-Rahimi et al.) [30]. The algorithm detected caries in 129 teeth, while the researchers identified this disease in 45 teeth. The number of decay-free teeth was 991 for the AI software and 1075 for the researcher, respectively. The calculated sensitivity of 75% was higher than in the preliminary study from 2023, where the AI framework was used to detect caries in DPRs and achieved 55.4% [32]. This performance metric was also higher compared to the study that used a deep convolutional neural network (CNN) to detect caries, which had a sensitivity of 44.5% [33]. However, it was lower than the 84% sensitivity achieved in identifying dentin caries in bitewings [34]. This difference may be attributed to bitewing radiographs being superior to DPRs for decay detection [35]. A positive correlation with false-positive results and a negative correlation with false-negative results in identifying teeth affected by caries suggest the subtle influence of the presence of a primary tooth in the assessment. It, therefore, can be assumed that a radiological image suggesting decay in a primary tooth may be interpreted by the AI algorithm as if it were in a permanent tooth.

### 4.2. Missing Teeth

Identically directed correlations are observed when assessing the presence of a permanent tooth in a given position in the oral cavity. The presence of a deciduous tooth increases the chance that the algorithm misidentifies a permanent tooth in a specific location. The absence of a deciduous tooth has the opposite effect, i.e., it increases the risk that the AI omits a permanent tooth. It should be emphasized, however, that these correlations are very subtle.

The results regarding missing teeth can be compared with four other systematic reviews that also cover teeth identification and numbering by AI models [26,36,37,38]. In the current study, the specificity and accuracy values were high (above 85%) but still lower than in the studies included in the abovementioned systematic reviews. The algorithm identified more missing teeth (96) than the researcher (87). A very high specificity (99.4%) in detecting missing teeth also occurred in the study by Zhu et al. [32]. However, a significant difference was observed in the sensitivity and precision, which in our study are very low (approximately 14%), while in the studies by Khanagar et al. and Umer et al., they were on average above 95% [26,38]. A frequently noticeable error of the algorithm was the failure to identify the absence of third molars, which are usually noticeable in a DPR at the age of approximately 10–12 years.

### 4.3. Filled Teeth

The sensitivity, specificity, and accuracy of detecting dental fillings in DPRs in this study were high, reaching over 87%. The number of teeth with dental fillings identified was 76 for the algorithm and 33 for the researcher. There are two other studies assessing the reliability of AI evaluation in panoramic radiographs using a deep convolutional neural network that incorporates U-Net-like and Mask R-CNN models for segmentation and diagnosis [33,39]. In both, sensitivity and specificity were comparable, approximately 85% and 96%, respectively [33,39]. A low precision of detecting not only fillings but also dental caries and missing teeth means that the algorithm returns a lot of false positives.

For caries, a weak correlation of false-positive results with the presence of a primary tooth in a given position in the oral cavity is observed. However, there is no such correlation for the assessment of dental fillings. Artificial intelligence algorithms are trained to detect based on the appearance of the radiological image, which in the case of fillings, most often means a strong radiological hyperdense area. They should be differentiated, among other things, from the overlap of images of two adjacent teeth. It was noticed that sometimes the algorithm classified orthodontic fixed appliances such as brackets or molar bands as fillings.

Reducing the number of false positives and thus improving precision can be achieved by further training of the algorithm, in particular, undertaking training on mixed dentition. Alternatively, it should be considered to limit the evaluation of restorations in DPRs in favor of dedicated bitewing radiographs.

### 4.4. Root Canal Fillings and Endodontic Lesions

The number of root canal filling (3) and endodontic lesion (30) cases identified was too small to discuss these calculated indicators. It was impossible to calculate the sample size for these variables, and the one used appeared insufficient. Nevertheless, attention must be drawn to the overdetection of endodontic lesions by the AI, which probably resulted from incomplete root development. It manifests with radiolucency in DPRs, often strikingly similar to periapical periodontitis. In such cases, a trained professional evaluates the entire tooth and determines whether there are any radiological indications of typical co-occurring conditions, e.g., deep caries or fracture, which AI is currently incapable of. Therefore, in future work on the algorithmic assessment of the tooth chamber, root canals and periapical tissues, the possibility of linking the tooth crown state with the probability of periapical lesions should be considered. In addition, the proximity of the radiological image of unfinished root apex development to periapical periodontitis provides a basis for taking into account the patient’s age.

### 4.5. Limitations

This study was conducted using only one AI algorithm. Individual algorithms are trained differently and produce different performance metrics. Therefore, it is not possible to extrapolate the results obtained to the overall capabilities of AI in analyzing DPRs.

Panoramic radiographs from a single diagnostic imaging facility were included. The frequency of specific diagnoses depends, among other things, on the selection of the study population. This study included patients who underwent diagnostic imaging in a medium-sized city in central Europe, all Caucasians. Hence, future studies should consider involving various, or even better, multiple centers.

Due to the paucity of data from previous studies, the sample size was calculated only based on the missing teeth variable. The required sample sizes for the variables decay, filled, root canal filling, and endodontic lesion have not yet been determined. Since the reference studies showed the highest accuracy for the variable missing teeth, it is assumed that smaller samples would be sufficient to assess the accuracy for the remaining variables. This and future studies will serve to refine the required sample sizes.

Another limitation is the assumption of the infallibility of human judgment, as doctors who evaluate panoramic radiographs and radiographs in general are fallible [40]. This was a necessary simplification resulting from the need to provide reference outcomes. Every doubt was consulted to reduce the percentage of errors. However, DPR analyses performed by different specialists can differ and lead to inconsistencies. The accuracy of diagnoses can depend on the experience or fatigue level of the dentist. The same person may also interpret the same radiograph differently at various times.

Primary dentition was also not assessed in this study as the algorithm was trained on permanent teeth. In the examined material, most of the deciduous teeth were partially resorbed in preparation for natural exfoliation. The lack of assessment of primary teeth in ideal conditions should not affect the assessment of permanent teeth. Nevertheless, it has been shown that there is an over-detection of decay and an under-detection of missing teeth in the case of a primary tooth in a given position in the oral cavity.

### 4.6. Strengths

The strengths of this study lie in its detailed assessment of AI performance in a previously neglected age group. This study provides information on the specific challenges AI faces when assessing the developing dentition of children.

The distinctions of sensitivity, specificity, precision, and accuracy in AI assessments are not standard in similar studies. They highlight directions for further training of the algorithm to achieve reliability in correctly identifying the presence of permanent teeth, caries, and fillings.

### 4.7. Future Perspectives

Given the increasing performance indicators of algorithmic DPR assessment, there is a temptation to raise the bar. AI detects basic pathologies in standard DPRs to a fairly satisfactory extent. The AI-driven assessment of unusual images is still a challenge. The atypicality may result from imaging errors, anatomical disorders, pathology presence, and developmental age. According to the presented results, the assessment of permanent dentition in the DPRs of pediatric patients can be performed algorithmically, but it is subject to greater errors. As for primary teeth, if carried out at all, an erroneous evaluation of a tooth that the algorithm interpreted as permanent occurs. Hence, identifying primary teeth should be implemented, at least so as to ignore them when assessing mixed dentition. Ultimately, deciduous dentition should undergo the same evaluation as permanent ones.

Nevertheless, the algorithm that was tested performed well, even though it was not intended to assess mixed dentition. It achieved specificity and accuracy values in detecting caries and missing teeth that were similar to those achieved in the AI models reported in a recent systematic review [18].

## 5. Conclusions

The overall accuracy of artificial intelligence algorithms for evaluating permanent dentition in dental panoramic radiographs is notably lower for pediatric patients compared to adults or the general population. Although the assessments demonstrate high specificity, they suffer from low precision. Low precision, and therefore a higher number of false positives, is a leading characteristic of the algorithmic assessment of mixed dentition, regardless of the diagnosis tested.

The lowest accuracy and sensitivity in detecting the absence of a permanent tooth in a specific position within the oral cavity may be largely attributed to the misidentification of primary teeth as permanent teeth, underscoring the need for more refined algorithms that account for the unique dental characteristics of pediatric patients.

The single-algorithm and single-center designs, inability to calculate sample size for most variables, assumption of the infallibility of human assessments, and the impossibility of primary dentition assessment limited the study design. Therefore, AI algorithms should be trained in the assessment of primary teeth in mixed dentition and further similar studies should be conducted in different populations or in multiple centers.

## Figures and Tables

**Table 1:** Criteria for inclusion and criteria for exclusion.

	Inclusion Criteria	Exclusion Criteria
Population	DPRs of patients with incomplete development of permanent dentition	Incomplete development of third molars only
Intervention	Assessment of the presence and condition of each permanent tooth by an AI algorithm	Incomplete or erroneous results due to software or human error
Comparator	Assessment of the presence and condition of each permanent tooth by researchers	Not applicable
Outcomes	Variables regarding the condition of teeth: decay, missing, filled, root canal filling, endodontic lesion	Not applicable
Settings	DPRs from 2022–2023 from a single diagnostic imaging facility in Kielce, Poland	DPRs of a quality inconsistent with local regulations

**Table 2:** Performance metrics used in the study.

Performance Metric	Formula
Sensitivity	$\frac{TP}{TP+FN}$
Specificity	$\frac{TN}{TN+FP}$
Precision	$\frac{TP}{TP+FP}$
Accuracy	$\frac{TP+TN}{TP+FP+TN+FN}$

*TP*—true positive; *TN*—true negative; *FP*—false positive; *FN*—false negative

**Table 3:** Outcomes.

	Decay	Missing	Filled	Root Canal Filling	Endodontic Lesion
Index test negative	991	1024	1044	1117	1090
Reference test negative	1075	1033	1087	1119	1115
Index test positive	129	96	76	3	30
Reference test positive	45	87	33	1	5
True positive results (correctly identified)	34	13	29	1	2
True negative results (correctly excluded)	980	950	1040	1117	1087
False-positive results (overdiagnosed)	95	83	47	2	28
False-negative results (misdiagnosed)	11	74	4	0	3
Sensitivity	75.56% (95% CI 60.46% to 87.12%)	14.94% (95% CI 8.20% to 24.20%)	87.88% (95% CI 71.80% to 96.60%)	100.00% (95% CI 2.50% to 100.00%)	40.00% (95% CI 5.27% to 85.34%)
Specificity	91.16% (95% CI 89.30% to 92.79%)	91.97% (95% CI 90.14% to 93.55%)	95.68% (95% CI 94.29% to 96.81%)	99.82% (95% CI 99.36% to 99.98%)	97.49% (95% CI 96.39% to 98.32%)
Precision	26.36% (95% CI 21.73% to 31.57%)	13.54% (95% CI 8.35% to 21.22%)	38.16% (95% CI 31.22% to 45.62%)	33.33% (95% CI 11.13% to 66.63%)	6.67% (95% CI 2.25% to 18.17%)
Accuracy	90.54% (95% CI 88.67% to 92.19%)	85.98% (95% CI 83.81% to 87.96%)	95.45% (95% CI 94.06% to 96.59%)	99.82% (95% CI 99.36% to 99.98%)	97.23% (95% CI 96.09% to 98.11%)

CI—confidence interval.

**Table 4:** Correlations of primary teeth presence with false positives and false negatives.

	Decayed Teeth	Missing Teeth	Filled Teeth	Root Canal Filling	Endodontic Lesions
Correlation with false positives	0.20	0.07	0.00	−0.02	−0.04
Correlation with false negatives	−0.05	−0.13	−0.03	N/A	−0.03

N/A—not applicable.

## Data Availability

The raw data supporting the conclusions of this article will be made available by the authors on request.
